# Cholecystectomy and the risk for developing colorectal cancer and distal colorectal adenomas

**DOI:** 10.1038/sj.bjc.6600661

**Published:** 2003-01-28

**Authors:** E S Schernhammer, M F Leitzmann, D S Michaud, F E Speizer, E Giovannucci, G A Colditz, C S Fuchs

**Affiliations:** 1 Channing Laboratory, Department of Medicine, Brigham and Women's Hospital and Harvard Medical School, 181 Longwood Avenue, Boston, MA 02115, USA; 2 Ludwig Boltzmann-Institute for Applied Cancer Research, KFJ-Spital, Vienna, Austria; 3 Department of Nutrition, Harvard School of Public Health, Boston, MA, USA; 4 Department of Epidemiology, Harvard School of Public Health, Boston, MA, USA; 5 National Cancer Institute, Nutritional Epidemiology Branch, Rockville, MD, USA; 6 Department of Environmental Health, Harvard School of Public Health, Boston, MA, USA; 7 Harvard Center for Cancer Prevention, Boston, MA, USA; 8 Epidemiology Program, Dana Faber/Harvard Cancer Center, Boston, MA, USA; 9 Department of Adult Oncology, Dana-Farber Cancer Institute, Boston, MA, USA

**Keywords:** colon cancer, colon adenomas, gallstones, cholecystectomy, risk factors

## Abstract

Earlier work describes a modest association between cholecystectomy and the risk of colorectal cancer. We conducted a prospective study of 85 184 women, 36–61 years old, who had no history of cancer to evaluate whether known risk factors for colorectal cancer, including dietary history, that have not been controlled for in previous analyses can help explain the observed association. During 16 years of follow-up, 877 cases of colorectal cancer were documented and 1452 women who underwent endoscopy during the follow-up time were diagnosed with distal adenomas. After adjustment for age and other known or suspected risk factors, we found a significant, positive association between cholecystectomy and the risk of colorectal cancer (multivariate relative risk RR 1.21, 95% CI 1.01–1.46). The risk was highest for cancers of the proximal colon (RR 1.34, 95% CI 0.97–1.88) and the rectum (RR 1.58, 95% CI 1.05–2.36). However, we did not observe a significant association between cholecystectomy and distal colorectal adenomas. In this large prospective cohort study, a history of cholecystectomy appears to increase modestly the risk of colorectal cancer, even after adjustment for other colorectal cancer risk factors.

More than 60 epidemiologic studies have investigated a possible relation between cholecystectomy and colon cancer risk. However, the strength of any association remains uncertain because of the small size and retrospective design with a failure to control for potential confounders of most analyses. A meta-analysis of studies conducted through 1993 (Giovannucci *et al*, 1993) observed a modest increase in the risk of colorectal cancer following cholecystectomy, although the effect was restricted primarily to the proximal colon. Two subsequent meta-analyses (Bollschweiler *et al*, 1993; Reid *et al*, 1996) and three large prospective cohort studies (Ekbom *et al*, 1993; Goldbohm *et al*, 1993; Lagergren *et al*, 2001) also observed an increased risk of proximal cancers, particularly among women.

Most studies that examined the relation between gallstones or cholecystectomy and colorectal cancer did not adjust for other risk factors for colorectal cancer, including diet. Therefore, among those studies demonstrating a positive association, one cannot exclude potential confounding by dietary or other lifestyle factors that are jointly associated with gallstones and colorectal cancer.

For the relation between cholecystectomy and colorectal cancer to be adequately characterised, large cohort studies with a long duration of follow-up and adequate assessment of potential confounding factors are necessary. We therefore utilised data from a prospective cohort study among women to illuminate further the influence of gallstones and cholecystectomy on the risk for colorectal cancer and distal colorectal adenoma.

## METHODS

### Study cohort

The Nurses' Health Study was initiated in 1976, when 121 700 female registered nurses of age 30–55 and residing in the United States completed a mailed questionnaire on known or suspected risk factors for cancer and coronary heart disease (Willett *et al*, 1987). Every 2 years since then, we have mailed follow-up questionnaires to these women to update information on these risk factors and major medical events. In 1980, we expanded the questionnaire to include an assessment of diet.

### Data on cholecystectomy, gallstones, and other factors

The study participants provided information on gallstones (yes/no, year of diagnosis) and cholecystectomy (yes/no, year of diagnosis) in 1982. This information was updated biennially for gallstones until 1986 and for cholecystectomy until 1998. Other questions relevant to the association between cholecystectomy/gallstones and colorectal cancer provided information on the study participants' age, smoking history, height, weight, physical activity, aspirin use, menopausal status, postmenopausal hormone use, family history of colorectal cancer, and previous examination by colonoscopy or sigmoidoscopy and the indications of the procedure. In the 1980 semiquantitative food-frequency questionnaire, participants reported the average frequency of their consumption of specific foods and beverages and supplemental vitamins during the previous 12 months. The reproducibility and validity of these semiquantitative food-frequency questionnaires have been documented previously (Willett *et al*, 1985,^1990^).

### Population for analysis

We excluded nurses who had not answered the 1982 questionnaire. To control adequately for dietary factors, we further excluded women who left 10 or more items blank on the 1980 food-frequency questionnaire and women with implausibly high or low values for total energy intake. Women who reported previous cancer (except nonmelanoma skin cancer), ulcerative colitis, Crohn's disease, or a familial polyposis syndrome were also excluded. After these exclusions, 85 184 women remained eligible for follow-up from 1982 through 1998.

### Identification of cases of colorectal cancer or adenoma

For this analysis, the follow-up data were available for 96% of the total possible person-years through 1998. Most of the deaths in the cohort were reported by family members or the postal system in response to the follow-up questionnaire. In addition, we used the National Death Index, a highly sensitive method of identifying deaths among nonrespondents (Stampfer *et al*, 1984). We estimate that we have identified over 98% of the deaths in this cohort through these sources (Stampfer *et al*, 1984). On each questionnaire, we inquired whether colon cancer or rectal cancer had been diagnosed and, if it had been, requested the date of the diagnosis. When a woman (or next of kin) reported a diagnosis of colorectal cancer on a follow-up questionnaire, we asked for permission to obtain medical records and pathology reports related to this diagnosis. A study physician blinded to the woman's exposure and intake data reviewed all records and extracted data on the histologic type, anatomical location, and stage of the cancer. We included only cases of adenocarcinomas, and excluded carcinoma *in situ* from the analysis. This left 877 cases of invasive colorectal carcinoma.

The analysis of adenomas was restricted to women who were eligible for the analysis of cancer and who reported having undergone a colonoscopy or sigmoidoscopy during the study period. Because the review of medical records for cases of adenoma has been completed only through 1996, the study period for adenomas was from 1982 through 1996. After all exclusions, 26 005 women were eligible for this portion of the analysis. When a woman reported a diagnosis of a colorectal polyp, we obtained medical records and pathology reports. A study physician unaware of the risk-factor data reviewed all records and extracted data on the type, location, and size of the adenoma (Giovannucci *et al*, 1994). Because a sigmoidoscopic examination encompasses only the distal portion of the colon and rectum and because a substantial portion of those who underwent endoscopy had a sigmoidoscopy, we assessed only adenomas of the descending colon and rectum. Women with only adenomas proximal to the descending colon were not classified as having adenoma in this analysis. This left 1452 patients with adenomas of the distal colon and rectum.

### Statistical analysis

For each eligible study participant, person-years of follow-up were counted up from 1 June 1982 to the date of a diagnosis of colorectal cancer or death or until 31 May 1998, whichever came first. If no questionnaire was returned for a follow-up interval, the most recently recorded data were used for the subsequent interval. We used relative risk (RR) as the measure of association, defining RR as the incidence of colorectal cancer among study women who reported a history of gallstones or cholecystectomy divided by the corresponding rate among the women who had no history of gallstones or cholecystectomy. Mantel Haenszel summary RRs were calculated, adjusting for age in 5-year categories (Rothman and Boice, 1979). All statistical tests were two sided. Tests for trend across categories of exposure were calculated by treating the levels of exposure as a continuous, ordinal variable in the regression model. Cox proportional-hazards models were used to calculate RRs with adjustment for age, pack years of smoking more than 35 years in the past in quintiles; body-mass index (weight in kilograms divided by the square of the height in meters) in five categories (<21, 21–22.9, 23–24.9, 25–28.9, 29+); physical activity, MET-h/week (sum of the average time/week spent in each activity by its typical energy-expenditure requirements expressed in metabolic equivalents (METs, the caloric need per kilogram of body weight per hour activity, divided by the caloric need per kilogram per hour at rest)), in quintiles; regular aspirin use (≥2 *vs* <2 times per week); colorectal cancer in parent or sibling (yes or no); screening endoscopy during the study period (yes or no); consumption of beef, pork, or lamb as a main dish (<1 serving per month, 1–3 per month, 1 per week, 2–4 per week, or ≥5 per week); current alcohol consumption status (abstinent, history of greatly reduced consumption, or <15, 15–30, or >30 g day^−1^); quintiles of total energy intake, use of postmenopausal hormones (never, past user <5 years, past user 5 years, current <5 years, current 5 years); menopausal status (yes or no); height in seven categories (⩽150, 151–155, 156–160, 161–165, 166–170, 171–175, 176–180, >180 cm); and diabetes (yes or no). For all factors, indicator variables were created for missing values and included in the analyses. We conducted stratified analyses to determine whether the influence of a history of cholecystectomy was modified by other risk factors for colorectal cancer. Tests for the homogeneity of risk estimates across strata were based on a weighted sum of the squared deviations of the stratum-specific log-odds ratios from their weighted mean (Rothman and Greenland, 1998).

## RESULTS

In 1982, a history of cholecystectomy was reported by 7. 7% of the 85 184 women who were eligible for analysis (14.9% by 1998). Women with a history of cholecystectomy were slightly older and slightly more likely to be postmenopausal than women without such a history (Table 1

**Table 1:** Baseline characteristics of the study cohort^a^ according to a history of cholecystectomy among 85 184 women in the NHS

	**History of cholecystectomy (1986)**	
**Characteristics**	**No**	**Yes**
Number of individuals	78 515	6669
Median age (years)	48.6	50.8
Current smoker (%)	26.8	30.2
Pack-years (>35 years in the past)^b^	3.0	3.4
Body-mass index ⩾30 (%)^c^	9.9	23.8
Physical activity, >once per week (%)	42.2	40.7
Regular aspirin use (⩾2 per week) (%)	24.1	29.8
Colorectal cancer in parent or sibling (%)	8.0	8.4
Screening endoscopy (%)	5.3	7.7
Intake of beef, pork, or lamb as main dish (2–4 servings per week) (%)	30.7	29.7
Alcohol intake (g day^−1^)	6.5	4.7
Height in inches	64.4	64.4
Regular multivitamin use (%)	37.9	34.9
Total energy intake	1567.0	1558.2
Postmenopausal in 1982 (%)	40.9	43.9
Ever use of postmenopausal hormones (%)^d^	10.3	13.1
Vitamin D intake (IU day^−1^)	277.1	266.9
Calcium intake (mg day^−1^)	701.1	701.2
Diabetes (%)	2.4	6.3

a All values other than age have been directly standardised according to the age distribution of the cohort.

b Pack-years were calculated for current smokers only.

c The body-mass index is weight in kilograms divided by the square of height in meters.

d Among postmenopausal women only.

). They were also more likely to be obese, smoke cigarettes, and use aspirin regularly, but they consumed less alcohol. Furthermore, women who reported cholecystectomy were more likely to have diabetes and undergone a screening endoscopy.

During 16 years of follow-up (1 308 490 person-years), 877 women were diagnosed with colorectal cancer. Among these 877 participants with colorectal cancer, 195 (22.2%) had previously reported a history of cholecystectomy.

In age-adjusted and multivariate analyses based on 16 years of follow-up, a history of cholecystectomy was positively associated with the risk of colorectal cancer (age-adjusted RR 1.21, 95% confidence interval (95% CI) 1.01–1.46). This risk associated with cholecystectomy was greater for proximal colon and rectal cancers ( Table 2

**Table 2:** Relative risk of colorectal cancer according to history of cholecystectomy among 85 184 women of the NHS^a^

	**Cholecystectomy**	
**Subsite**	**Number**	**RR (95% CI)**
Proximal colon cancer^b^		
ARR	46	1.34 (0.97–1.84)
MRR		1.35 (0.97–1.88)
Distal colon cancer^b^		
ARR	28	0.93 (0.63–1.38)
MRR		0.95 (0.64–1.43)
All colon cancer		
ARR	74	1.15 (0.90–1.47)
MRR		1.17 (0.91–1.51)
Rectal cancer		
ARR	32	1.69 (1.14–2.50)
MRR		1.58 (1.05–2.36)
Total colorectal cancer		
ARR	133	1.21 (1.01–1.46)
MRR		1.19 (0.98–1.44)

a ARR, age-adjusted relative risk; MRR, multivariate relative risk; RR, relative risk; CI, confidence interval.

b Proximal colon denotes the segment from the cecum to the splenic flexure, and distal colon denotes the segment from the splenic flexure to the rectosigmoid junction. The numbers of colon and rectal cancers may not be equal to the total number of colorectal cancers because in some cases the specific site of the cancer was unknown.

). Adjustment for other known or suspected risk factors did not substantially alter the estimates (multivariate RR 1.19, 95% CI 0.98–1.44). A history of gallstones was associated with similar risks (data not shown).

The diagnosis of gallstones or the need for cholecystectomy might have been triggered by early symptoms associated with colorectal cancer. To address this question, we repeated our analysis after excluding the first 6 years of follow-up after the first report of cholecystectomy (1982–1988). We analysed the relation between a history of cholecystectomy as assessed in 1982 and the risk of colorectal cancer from 1988 through 1996. Despite the 6-year latency period, a history of cholecystectomy was associated with a similar increase in the risk of colorectal cancer (multivariate RR 1.22, 95% CI 0.95–1.56).

To evaluate possible distortions of our findings by higher rates of screening endoscopy among women who reported a history of cholecystectomy, we repeated our analysis after stratification according to whether or not women had undergone screening endoscopy during the study period. Among women who had undergone screening endoscopy, the risk of colorectal cancer associated with gallstones or cholecystectomy was not significantly different when compared to women who had not undergone screening (RR 1.67, 95% CI 0.61–4.53 *vs* RR 1.19, 95% CI 0.96–1.46, respectively).

We also examined the association between cholecystectomy and colorectal cancer stratified according to risk factors potentially common to both colorectal cancer and gallstones. We found no consistent change in the relation between cholecystectomy and colorectal cancer in any subgroup defined by body-mass index, regular physical activity, intake of fiber, total fat, animal fat, and vegetable fat (data not shown).

We further investigated the possibility that the excess risk of colorectal cancer associated with cholecystectomy was the result of a detection bias caused by closer surveillance of people with a history of gallstones or cholecystectomy. When we excluded from the analysis women whose cancer may have been detected incidentally or only by screening (Duke's stages A and B) and restricted the outcome to more advanced lesions, the RR for colorectal cancer was not materially altered (RR 1.21, 95% CI 0.89–1.65).

We assessed the relation between cholecystectomy and the risk of distal colorectal adenomas among 26 005 women who reported having undergone colonoscopy or sigmoidoscopy between 1982 and 1996. There was no elevation of risk for distal colorectal adenoma with a history of cholecystectomy, even when we restricted our analysis to women who had undergone endoscopy before 1982 and were found at that time to be free of polyps ( Table 3

**Table 3:** Relative risk of distal colorectal adenoma according to history of cholecystectomy among 26005 women of the NHS (1452 cases of distal colorectal adenoma)^a^

	**No. of cases/total no. of women**	**Multivariate RR (95% CI)**
*All women with adenomas*		
All adenomas	1452/26 005	1.05 (0.88–1.26)
Small adenomas	780	1.06 (0.83–1.35)
Large adenomas	590	1.03 (0.78–1.36)
		
*Women with adenomas and previously negative endoscopic results*^b^		
All adenomas	332/7182	1.06 (0.74–1.52)
Small adenomas^c^	188	1.04 (0.65–1.67)
Large adenomas^d^	122	0.91 (0.49–1.69)

a RR, relative risk; CI, confidence interval.

b Women who underwent endoscopy before 1982 with negative results.

c Small sigmoid/rectal adenomas (<1 cm).

d Large sigmoid/rectal adenomas (⩾1 cm)

).

Finally, we examined the relation between the time since cholecystectomy and the risk of both colorectal cancer and distal colorectal adenomas (Table 4

**Table 4:** Relative risk of colorectal cancer and distal colorectal adenoma by time interval after cholecystectomy^a^

		**Multivariate RR (95% CI)**			
**Time interval**	**No. of women**	**Proximal colon**	**Distal colon**	**Rectum**	**Colon and rectum combined**
*Colorectal cancer*					
No cholecystectomy	744	1.00	1.00	1.00	1.00
Time interval after cholecystectomy (yr)					
0–4	19	2.28 (1.29–4.03)	0.19 (0.03–1.36)	1.19 (0.43–3.25)	0.98 (0.62–1.56)
5–9	17	0.74 (0.28–2.01)	1.17 (0.51–2.66)	1.04 (0.33–3.29)	0.98 (0.60–1.58)
10–14	28	1.45 (0.71–2.97)	1.50 (0.70–3.22)	2.71 (1.39–5.30)	1.49 (1.01–2.18)
15–19	30	1.54 (0.80–2.95)	1.06 (0.49–2.29)	1.37 (0.60–3.15)	1.39 (0.96–2.02)
20–24	15	0.83 (0.31–2.25)	0.27 (0.04–1.91)	1.63 (0.58–4.60)	0.98 (0.58–1.64)
25+	24	1.17 (0.54–2.54)	1.55 (0.67–3.58)	1.52 (0.60–3.83)	1.25 (0.82–1.89)
					
*P* value for trend^b^		0.18	0.54	0.30	0.94
					
*Distal colorectal adenoma*					
			**Large adenomas**	**Small adenomas**	**All adenomas combined**
No cholecystectomy	1308		1.00	1.00	1.00
Time interval after cholecystectomy (yr)					
0–4	28		1.34 (0.76–2.36)	1.16 (0.76–2.36)	1.18 (0.80–1.75)
5–9	12		1.55 (0.72–3.33)	0.82 (0.34–2.01)	1.07 (0.60–1.93)
10–14	37		0.78 (0.44–1.40)	1.22 (0.80–1.85)	1.00 (0.71–1.40)
15+	67		0.94 (0.63–1.42)	1.04 (0.73–1.48)	1.06 (0.82–1.37)
					
*P* value for trend^b^			0.78	0.53	0.90

a RR, relative risk; CI, confidence interval.

b *P* value (Wald test) for continuous linear term, only among women who had a cholecystectomy. The numbers of colon and rectal cancers may not be equal to the total number of colorectal cancers because in some cases the specific site of the cancer was unknown.

). Among individuals with a cholecystectomy up to 4 years in the past, the multivariate RR for cancer of the right colon was 2.28 (95% CI 1.29–4.03), whereas for those with cholecystectomy more than 24 years in the past, the RR was 1.17 (95% CI 0.54–2.54). Within all analyses of colorectal cancer, we did not observe any statistically significant positive or inverse trend in risk. Similarly, we observed no significant trend with total distal colorectal adenomas, although we did observe a significant positive trend for rectal adenomas following a cholecystectomy (*P*=0.02).

## DISCUSSION

In this large, prospective cohort study, we found an elevated risk for colorectal cancer associated with a history of gallstones or cholecystectomy. The risk was highest for cancers of the proximal colon (RR 1.36, 95% CI 1.00–1.86) and rectum (RR 1.64, 95% CI 1.12–2.39). We did not, however, observe the same association between gallstones or cholecystectomy and distal colorectal adenomas. We did detect a slight nonsignificant increase of risk according to time interval between cholecystectomy and cancer of the rectum in this cohort, as well as a significant trend for rectal adenomas with increasing time following a cholecystectomy.

Abnormal bile acid metabolism may facilitate the development of gallstones and may also increase the risk of colon cancer. The composition of bile acid is altered among patients who have undergone a cholecystectomy, with a high portion of secondary bile acids in their pool (Malagelada *et al*, 1973). Furthermore, the amount of fat in the stool may increase after cholecystectomy (Brydon *et al*, 1982). The continuous exposure of the gut to higher levels of metabolic products, such as biliary acids, undigested fat, and other end products of the colonic microflora (e.g. 3-methylindole), in cholecystectomised patients (Zuccato *et al*, 1993) might further elevate colon cancer risk. Animal models suggest that colon cancer risk increases with higher levels of secondary bile acids (Morvay *et al*, 1989).

Prior evidence for the hypothesis that a history of gallstones or cholecystectomy is related to the risk of colorectal cancer has been inconclusive. A meta-analysis of five either small (Wu *et al*, 1987) or population-based cohort studies (Rundgren and Mellstrom, 1983; Adami *et al*, 1987; Gudmundsson *et al*, 1989; Nielsen *et al*, 1991) and 33 case–control studies demonstrated a significantly increased combined RR for colorectal cancer following cholecystectomy of 1.24 (95% CI 1.10–1.40) for women (Giovannucci *et al*, 1993). The cohort studies alone showed no association between cholecystectomy and colorectal cancer in this meta-analysis (RR 1.01). The case–control studies, however, suggested an increased risk of right-sided colon cancer (RR 1.88) with a minimally increased risk for left-sided colon cancer (RR 1.09). Two other meta-analyses reported similar results, with a particular elevation in the risk of proximal colon cancers (RR 1.52 and 1.86, respectively) (Bollschweiler *et al*, 1993; Reid *et al*, 1996). Only three large prospective cohort studies (Ekbom *et al*, 1993; Goldbohm *et al*, 1993; Lagergren *et al*, 2001) have examined the relation between cholecystectomy and colorectal cancer. All of these studies found an elevated risk for proximal colorectal cancer among women (RR 1.24, 95% CI 1.03–1.48 and RR 1.89, 95% CI 1.04–3.42 (Ekbom *et al*, 1993; Goldbohm *et al*, 1993)) and women and men combined (RR 1.16, 95% CI 1.08–1.24). The risk of total colon (Ekbom *et al*, 1993) or colorectal cancer (Goldbohm *et al*, 1993) associated with cholecystectomy appeared to increase significantly with increasing time following cholecystectomy. The RRs were 1.54 (95% CI 1.03–2.22) and 1.66 (*P*-value for trend 0.03) for women with 15+ and 24+ years, respectively, since cholecystectomy. No significant trend was seen in the third study (Lagergren *et al*, 2001).

Fewer studies have examined the influence of cholecystectomy or gallstones on the risk of colorectal adenomas. Neugut *et al*, (1991) did observe an increased risk of adenomas among women who had undergone cholecystectomy; the effect of cholecystectomy appeared to increase more than 10 years after the procedure. We did not observe a significant association between gallstones or cholecystectomy and distal colorectal adenomas, although we did find a significant increase in risk of rectal adenomas with increasing time interval after cholecystectomy. Nonetheless, since our data on adenomas were restricted to lesions within the reach of the flexible sigmoidoscope, we were unable to examine the relation with proximal adenomas.

The prospective nature of our study precluded recall bias and the need of next-of-kin respondents. It is therefore unlikely that our results were due to biased assessment of a history of gallstones or cholecystectomy or colorectal cancer and adenomas. Moreover, to avoid misclassification of exposure, we updated reports of cholecystectomy and gallstones biennially. Differential follow-up is unlikely to have had a material influence, since follow-up was nearly complete for both fatal and nonfatal end points. Furthermore, the identification of deaths is highly accurate in this cohort (Stampfer *et al*, 1984). Detection bias also appears unlikely, since the association between a history of gallstones or cholecystectomy and colorectal cancer persisted when the analysis was restricted to advanced cases of colorectal cancer (i.e. Duke's stages C, D, and fatal cases).

Residual confounding remains yet another possible explanation for the positive association between gallstones or cholecystectomy and colorectal cancer risk, and may have explained the positive findings in prior studies. However, in the current study, the association with rectal, proximal colon, or total colorectal cancer was not altered after adjustment for known or suspected risk factors for colorectal cancer. We further stratified the analyses by screening endoscopy and did not observe any substantial differences in the relation between gallstones/cholecystectomy and colorectal cancer.

In conclusion, our study demonstrates a modest though statistically significant increased risk of colorectal cancer following gallstones or cholecystectomy, even after adjusting for other known or suspected risk factors for colorectal cancer. Consistent with other studies the risk appeared slightly greater for cancers of the proximal colon and rectum. We therefore cannot refute the hypothesis that a history of gallstones or cholecystectomy may promote colorectal carcinogenesis.
